# An Integrating Immune-Related Signature to Improve Prognosis of Hepatocellular Carcinoma

**DOI:** 10.1155/2020/8872329

**Published:** 2020-11-03

**Authors:** Rui Zhu, Wenna Guo, Xin-Jian Xu, Liucun Zhu

**Affiliations:** ^1^Department of Mathematics, Shanghai University, Shanghai 200444, China; ^2^School of Life Sciences, Shanghai University, Shanghai 200444, China; ^3^School of Life Sciences, Zhengzhou University, Zhengzhou, Henan 450001, China

## Abstract

Growing evidence suggests that the superiority of long noncoding RNAs (lncRNAs) and messenger RNAs (mRNAs) could act as biomarkers for cancer prognosis. However, the prognostic marker for hepatocellular carcinoma with high accuracy and sensitivity is still lacking. In this research, a retrospective, cohort-based study of genome-wide RNA-seq data of patients with hepatocellular carcinoma was carried out, and two protein-coding genes (GTPBP4, TREM-1) and one lncRNA (LINC00426) were sorted out to construct an integrative signature to predict the prognosis of patients. The results show that both the AUC and the C-index of this model perform well in TCGA validation dataset, cross-platform GEO validation dataset, and different subsets divided by gender, stage, and grade. The expression pattern and functional analysis show that all three genes contained in the model are associated with immune infiltration, cell proliferation, invasion, and metastasis, providing further confirmation of this model. In summary, the proposed model can effectively distinguish the high- and low-risk groups of hepatocellular carcinoma patients and is expected to shed light on the treatment of hepatocellular carcinoma and greatly improve the patients' prognosis.

## 1. Introduction

Liver cancer is one of the dangerous diseases threatening human health [1]. It remains to be the second leading cause of mortality in males and sixth in females. Meanwhile, the prevalence of liver cancer was about 780000 per year, while the deaths were 745500 per year. Hepatocellular carcinoma (HCC) is the primary histopathological type, accounting for 90% of the liver cancer cases. It is characterized by high malignancy, rapid progression, and proneness to metastasis and relapse [2, 3]. The recurrence rate of the HCC following surgery was as high as 60-70%, with a 5-year overall survival of only 4%, showing a great risk for human health [4–6].

Despite the existence of multiple therapeutic strategies for HCC treatment, efficacy displays remarkable divergence among individual patients [7]. Surgical resection and liver transplantation remain the main cure measures for the HCC, and the 5-year survival in few patients could be improved to 40-70% following surgery or liver transplantation, whereas relapse or metastasis following surgery occurs in about 80% of patients [8–10]. Patients who underwent ablation have fewer complications and quick recovery, while efficacy largely depends on the tumor size and metastatic status, showing no superiority over surgery in disease-free survival [11–13]. Due to this scenario, most HCC patients have a poor prognosis. Thus, the establishment of an effective risk stratification method is essential to improve the prognosis of the HCC patients [14–16].

Growing evidence suggests that the superiority of long noncoding RNAs and messenger RNAs could act as biomarkers for diagnosis, prognosis, and treatment [17]. For example, several studies have shown the potential importance of lncRNA and mRNA signatures as biomarkers for predicting the prognosis of non-small-cell lung cancer [18], breast cancer [19], colon cancer [20], and bladder cancer [21]. At present, some genes have already been reported to be the prognostic markers for the HCC [22–26]; for example, GSTM1 and GSTT1 gene polymorphisms are closely associated with familial inheritance of the HCC [27]. Zhang et al. reported chromosome 1p36.22 as the region of the HCC susceptibility gene [6]. Eun et al. analyzed the data of 377 tumor specimens and 21 normal tissues from TCGA database and uncovered the significant correlation of the NOX gene family with the patient's survival time and tumor metastasis [28]. Results revealed that FGFR4, IGF1R, and NEK2 are associated with the risk of HCC, as potential prognostic molecular markers [29–31]. Nevertheless, most of the mentioned genes performed poor accuracy and stability.

In this research, we contributed a three-gene model to distinguish the risk stratification of HCC patients based on TCGA database. The model shows good stability and high accuracy in both TCGA and GEO datasets and thus is expected to give the medical staff some ideas to improve the treatment effect of the HCC.

## 2. Materials and Methods

### 2.1. Data Source

Clinical information of HCC patients and the gene expression level (generated by RNA-seq) were downloaded from TCGA database (TCGA-LIHC) [32] and NCBI-GEO database (GSE14520). TCGA-LIHC dataset consists of 377 HCC specimens, among which 1 specimen had no survival information and 6 had no RNA expression data. The GSE14520 dataset contains 488 specimens. After removing 241 nontumor and 5 survival time-missing samples, the remaining 370 specimens in TCGA and 242 specimens in GSE14520 were used in this study. To avoid overfitting, 370 specimens in TCGA dataset were randomly allocated into the training dataset (187 specimens) and the validation dataset (TCGA validation dataset, 183 specimens). The age, gender, clinical stage, and tumor grade of the patients were also collected. Clinical stages were classified into stages I to IV based on the AJCC staging system, and the tumor grade was classified into G1 to G4 based on histologic characteristics [33] (Table 1, Supplementary Table 1). Moreover, the 242 specimens from GSE14520 were used to further evaluation.

### 2.2. Statistical Analysis

Cox proportional-hazards regression model [34] was used to analyze the correlation between survival time and genes. The Kaplan-Meier method [35], log-rank test [36], AUC [37], and C-index were employed to verify the performance of a classifier model. In order to determine the optimal prognosis model construction scheme, the lasso, random forest, relative expression ordering (REO), and mRMR methods [38] were used to screen the features and the corresponding classification models were then constructed by multivariate Cox regression. Finally, the analysis scheme combining traversal and Cox regression with the best performance in the risk stratification of HCC patients was chosen. Additionally, in this study, the expression of RNA-seq was normalized by log2 (FPKM-UQ+1), of which FPKM-UQ [39, 40] is a modified version of the FPKM [41, 42] (the values of FPKM-UQ were provided by TCGA database).

### 2.3. Calculation Method

All Cox regression, KM, and concordance index analyses were performed using R and Perl scripts. ROC analysis involves traversing all possible combinations and calculating the area under the receiver operating characteristic curve (AUC). Because of the large number of combinations, it is time-consuming to calculate AUC with the R package. Therefore, a new parallel Perl script which was a lot more efficient was developed to complete this step (Supplementary Source code). Furthermore, the correlation between gene expression and immune infiltration was carried out by using TIMER, and the functional enrichment analysis was carried out by DAVID.

## 3. Results

### 3.1. Construction of the RNA Prognostic Biomarker Model

In order to find biomarkers universally applicable for most patients, the treated FPKM-UQ value of each retained RNA must not be zero in at least half of the HCC specimens and so 26880 RNA data were recruited. Subsequently, the univariate Cox regression was recruited to analyze the survival time of the patients with the expression levels of 26880 individual RNAs. Results revealed 415 RNAs as candidates for the next-step model establishment, which were screened by the Wald test with *P* values less than 0.001, representing a significant correlation with the survival time.

After 415 candidates were selected out, the models were first constructed by multivariate Cox regression in the training dataset and then the model with the highest AUC from all of the possible combinations of one to three genes was chosen as the final prognostic biomarker model, enclosing 3 RNAs (ENSG00000238121, ENSG00000107937, and ENSG00000124731). The calculation equation was as follows:

(1) $$\begin{array}{c} \text{Risk score}=\left(-0.2466*E_{\text{ENSG}00000238121}\right)+\left(0.7675*E_{\text{ENSG}00000107937}\right)+\left(0.1726*E_{\text{ENSG}00000124731}\right). \end{array}$$

*E*_ENSG00000238121_, *E*_ENSG00000107937_, and *E*_ENSG00000124731_ were accordingly referred to the RNA levels of ENSG00000238121, ENSG00000107937, and ENSG00000124731. The gene information are listed in Table 2.

### 3.2. Property Evaluation of the Model Enclosed in TCGA Training and Validation Datasets

**Figure 1:**
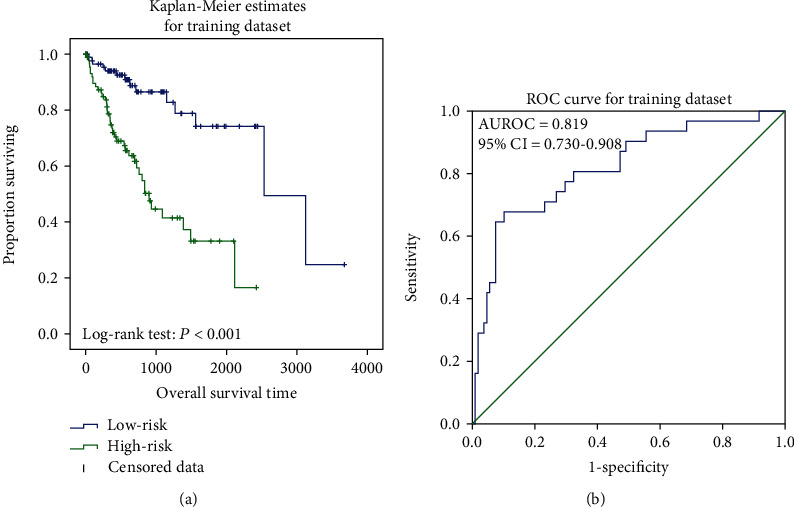
Kaplan-Meier analysis for TCGA training dataset. It showed the significant differences between the two curves (*P* < 0.001) (a). ROC curve of survival prediction. The AUC was 0.819 (95% CI = 0.730-0.908) (b).

**Figure 2:**
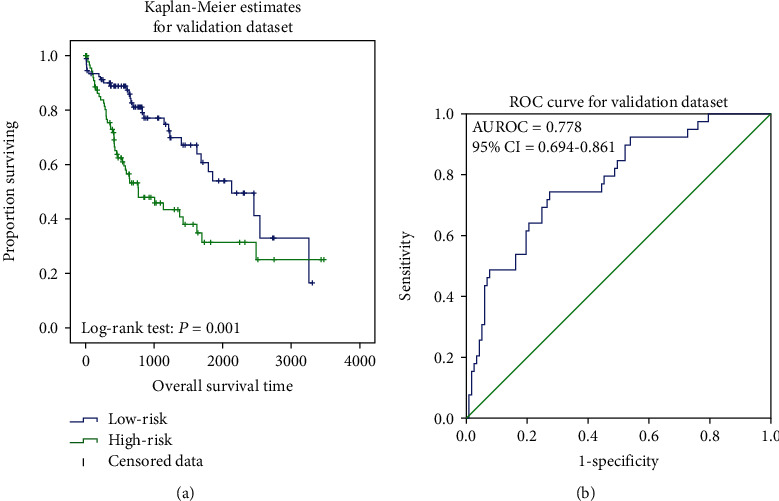
Kaplan-Meier analysis for TCGA validation dataset. It showed the significant differences between the two curves (*P* = 0.001) (a). ROC curve of survival prediction. The AUC was 0.778 (95% CI = 0.694-0.861) (b).

Patients were evenly separated into two groups based on their risk scores. In both the training and validation datasets, the mean survival time of the low-risk group was higher (847 vs. 597 days, 1015 vs. 747 days). The KM analysis and log-rank test results also showed the significant differences between the two groups (*P* ≤ 0.001) (Figures 1(a) and 2(a)). Meanwhile, the AUC values were 0.819 (95% CI of 0.730-0.908, *P* < 0.001) and 0.778 (95% CI of 0.694-0.861, *P* < 0.001) in the training (Figure 1(b)) and validation (Figure 2(b)) datasets, and the concordance index (C-index) values in those two datasets were 0.760 (95% CI of 0.692-0.828, *P* < 0.001) and 0.685 (95% CI of 0.622-0.749, *P* < 0.001), reflecting the high accuracy of the RNA prognostic model.

### 3.3. Identifying the Prognostic Performance of the Model in Divergent Groups of Clinical Characteristics

**Figure 3:**
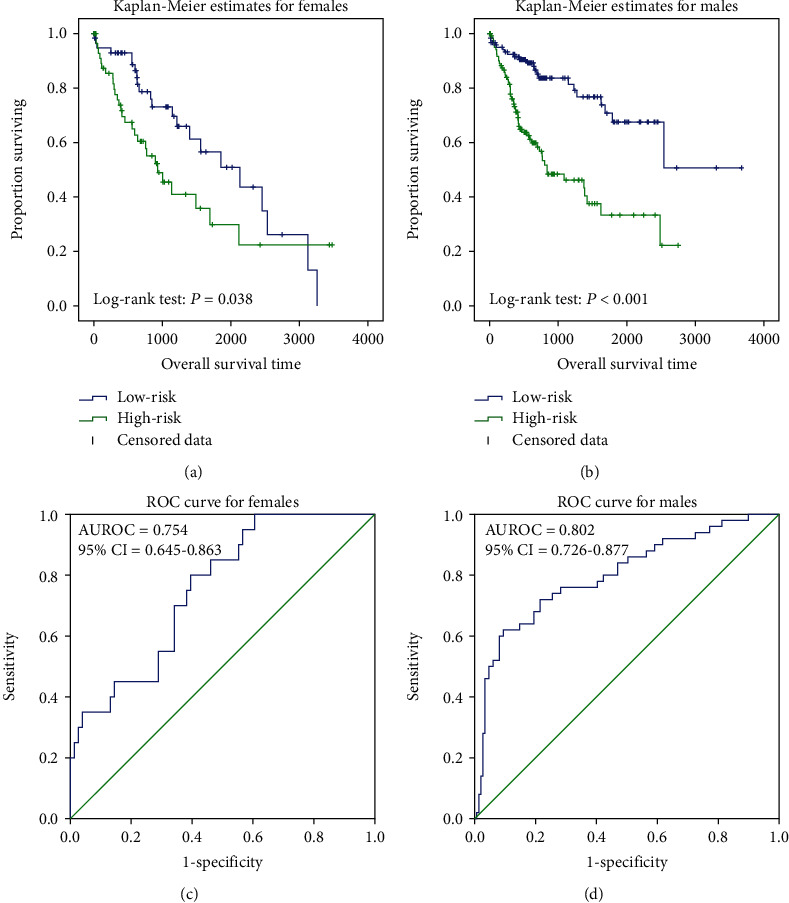
Kaplan-Meier analysis in different gender groups. It showed the significant differences between the two curves in different gender groups (*P* = 0.038, *P* < 0.001) (a, b). ROC curve of survival prediction in different gender groups. The AUC were 0.754 (95% CI = 0.645-0.863) and 0.802 (95% CI = 0.726-0.877) (c, d).

Patients' gender, clinical stage, and tumor grade can be considered prognostic factors, which affected the initiation and development of cancers at certain stages. According to these 3 clinical characteristics, 370 HCC patients were reallocated from the groups to further validate the high feasibility of the RNA prognostic biomarker model. Firstly, patients were regrouped by gender, and results showed that the survival duration was significantly (*P* < 0.001, *P* = 0.038) different in two risk groups in both the gender groups (Figures 3(a) and 3(b)). The AUC values were 0.802 and 0.754, and the C-index values were 0.733 and 0.646, respectively, for the male and female groups (Figures 3(c) and 3(d)). Then, the validation model was eligible for HCC patients in divergent clinical stages and tumor grades. As shown in Supplementary Figure 1, Supplementary Figure 2, and Supplementary Table 2, except for stage II, the RNA prognostic biomarker model was eligible for all other groups for predicting the patient survival status, with high prediction accuracy.

### 3.4. Property Evaluation in the Cross-Platform GEO Validation Dataset

To further access the applicability of our model, a cross-platform dataset (GSE14520) from the GEO dataset was recruited. The Kaplan-Meier analysis in this dataset also proved that the survival time of the low-risk group is significantly longer (*P* = 0.009). The AUC was 0.641 (95% CI of 0.561-0.721, *P* = 0.003) (Supplementary Figure 3), and the C-index was 0.592 (95% CI of 0.538-0.647, *P* < 0.001). These results suggested that our model is efficient in predicting the survival of patients.

### 3.5. Analysis of the Expression Pattern of RNA Prognostic Biomarkers

**Figure 4:**
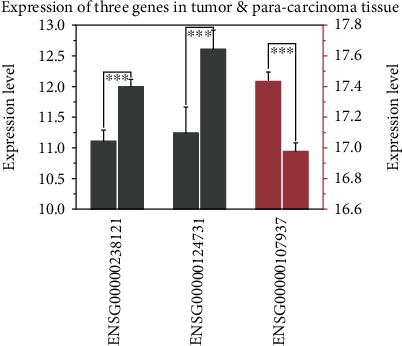
Histogram of differential expression of 3 RNA prognostic biomarkers between the cancerous and paracancerous tissues. The *X*-coordinate stands for the name of biomarkers, the left *Y*-coordinate (in grey) represents the gene expression level of ENSG00000124731 and ENSG00000238121, and the right *Y*-coordinate (in red) represents the gene expression level of ENSG00000107937.

We analyzed the expression pattern of these 3 RNAs contained in our model to find their function in the occurrence and development of the HCC. One notices significant differences in the expression levels of the 3 RNA prognostic biomarkers between the cancerous and paracancerous tissues, with *P* < 0.001 for all the 3 biomarkers (Figure 4) which suggested that the 3 RNAs might be crucial in the development of HCC.

Meanwhile, differential RNA expression analysis was conducted in different clinical stages (Supplementary Figure 4). The expression levels of the 3 RNAs showed a clearly ascending or descending trend along with the rise of clinical stages under most of the conditions.

### 3.6. The Correlation of the Prognostic Biomarker Expression and Immune Infiltration Level

**Figure 5:**
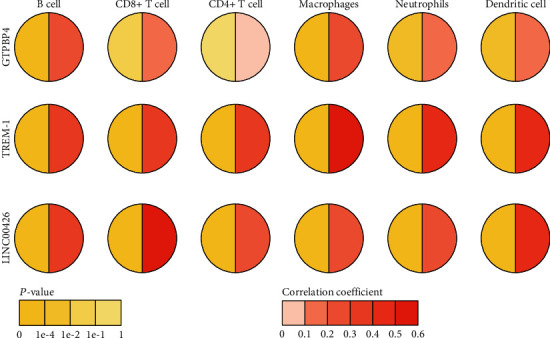
Correlation between the expression of biomarkers from RNA prognostic biomarker combination and immune cell infiltration level in hepatocellular carcinoma. The *X*-coordinate and *Y*-coordinate stand for the name of the immune cell and biomarker, respectively. Circle symbols represent the one-to-one correlation, the left half of the circle represents the *P* value, and the right half of the circle represents the correlation coefficient.

The infiltration level of tumor-infiltrating immune cells has an important influence on cancer treatment efficacy and patient prognosis. The results showed that the expression level of GTPBP4 was significantly correlated with the infiltration level of B-cells and macrophages (correlation coefficient > 0.2, *P* < 1.0*e* − 04); the expressions of TREM-1 and LINC00426 were significantly correlated with all these kinds of cells (correlation coefficient > 0.2, *P* < 1.0*e* − 04) (Figure 5, Supplementary Figure 5). These results suggested that the 3 RNA prognostic biomarkers may be linked to tumor immunity.

### 3.7. The RNA Expressions of Prognostic Biomarkers in Cancer Cell Lines

**Figure 6:**
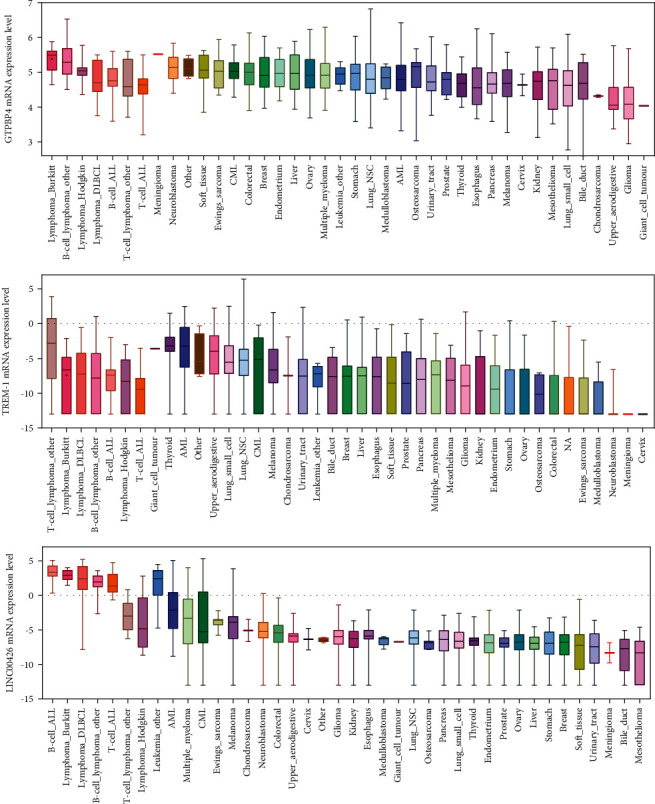
The box plots of the expression level of 3 prognostic biomarkers in the CCLE database. The *X*-coordinate and *Y*-coordinate stand for the name of the cell line and the expression level of the gene, respectively. In total, 1457 cell lines from 40 different types were included.

The expression pattern of the three biomarkers in all types of cancer cell lines in the Cancer Cell Line Encyclopedia (CCLE) database was also studied. In total, 1457 cell lines from 40 different types were included. The results showed that three prognostic biomarkers had relatively high expression levels in immune-related cell lines (Figure 6).

### 3.8. The Subcellular Localization

The subcellular localization of RNA is critical to understanding the regulation and function of RNA. RNALocate [43], iLoc-lncRNA [44], and iLoc-mRNA [45] were used to retrieve and predict the subcellular localization of the 3 prognostic markers in this study. The results showed that all three markers can be localized in exosomes, which were widely distributed in various body fluids, affecting the physiological state of cells, and were closely related to the occurrence and progression of various diseases (Supplementary Table 3).

### 3.9. Gene Pathway Analysis

**Figure 7:**
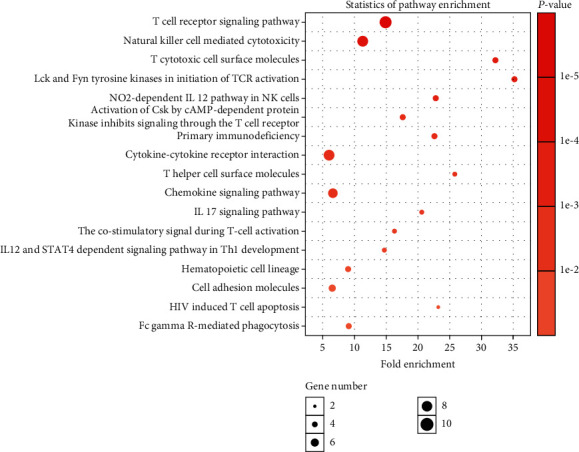
Seventeen enriched pathways related to genes that were coexpressed with 3 biomarkers from RNA prognostic biomarker combination for hepatocellular carcinoma. The *X*-coordinate and *Y*-coordinate stand for the fold enrichment and the name of the pathway, respectively.

The pathway analysis of related genes may reveal the biological processes known to be involved in cancer. Here, we first calculated the Pearson correlation coefficient (PCC) between the expression levels of all RNAs and that of the 3 RNAs in our model. After that, 98 genes, which were significantly correlated with the 3 RNA biomarkers in the model (*P* < 0.01 and PCC > 0.6), were chosen for further analysis. Then, the functional enrichment analysis of these 98 genes was carried out by DAVID [46, 47], yielding that these genes were primarily enriched in 17 pathways (Figure 7, Supplementary Table 4), most of which were relevant to the antigen-specific immunity. Nevertheless, immunity is closely related to tumor therapy efficacy. According to the physiological processes of recognizing and killing tumor cells by the immune system, the immunity relevant pathways were categorized as follows.

The first type of pathways was tumor recognition relevant in the immune system. In these pathways, a large number of cytokines played an essential role in recognizing the tumor that bridges antigen-presenting cells (*APCs*) and immune effector cells, such as the cell adhesion molecules (*CAMs*), cytotoxic T-cell surface molecules, cytokine-cytokine receptor interaction, and *NO2*-dependent *IL*-*12* pathway in natural killer (*NK*) cells.

The second type of pathways was immune cell differentiation relevant. In these pathways, the immune cells, upon stimulation by cytokines, ultimately differentiate into effector cells and kill tumor cells, such as the hematopoietic cell lineage, chemokine signaling pathway, *IL*-*17* signaling pathways, and *IL*-*12* and *STAT4*-dependent signaling pathway in *Th1* cell development.

The third type of pathways was immune cell activation and cytotoxicity relevant. The immune cell activation is a critical phase for immunological cytotoxicity towards tumor cells. The enrichment analysis displayed a large number of genes enriched in the T-cell activation pathways. Since T-cell is the primary cell involved in the tumor-specific immune response, it implied the reliability of our data. These pathways encompassed the T-cell receptor signaling pathway, *Lck* and *Fyn* tyrosine kinases in the initiation of *TCR* activation, activation of *Csk* by *cAMP*-dependent protein kinase inhibiting signaling through the T-cell receptor, T-cell receptor signaling pathway, T helper (*Th*) cell surface molecules, and costimulatory signal during T-cell activation. In addition, *NK* cells and *B*-cells, in this study, participated in tumor cytotoxicity induced by the immune cells during the effect phase, such as *NK* cell-mediated cytotoxicity and *FcγR*-mediated phagocytosis.

The last type of pathways was immune suppression relevant. Primary immunodeficiency, as shown in enrichment analysis, belongs to these pathways closely related to tumor immune tolerance.

### 3.10. Superiority of the RNA Prognostic Biomarker Model

**Figure 8:**
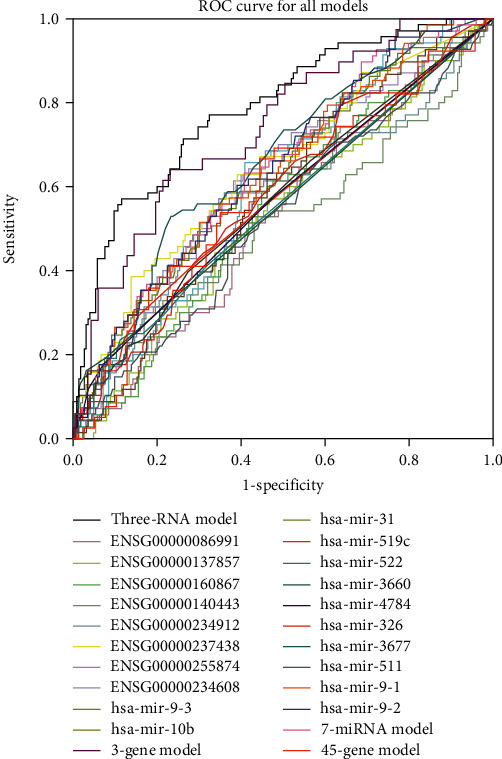
The AUC of the combination in comparison with other known biomarkers [22, 23, 28, 30, 31, 48–51] in the validation dataset of TCGA-LIHC. Different colors represent the ROC curves of different biomarkers, and the relationships between them are shown in the right area of the figure. The upper curve represents the larger AUC and better predictive performance. The ensemble gene IDs and miRNA names stand for the single prognostic biomarker models, while the others stand for the combined prognostic biomarker models. The three-RNA model (The black curve) is the model proposed by our study, showing a significantly higher AUC than that of the other biomarkers. Meanwhile, the 7-miRNA model, the 3-gene model, and the 45-gene model were provided by Zhang et al. [48], Li et al. [22], and Gillet et al. [23], respectively.

Some prognostic biomarkers for the HCC have also been reported. To validate the prediction superiority of the combination obtained by RNA prognostic biomarkers, molecular prognostic biomarkers from the references were chosen to compare with our RNA prognostic biomarkers, followed by the comparison of prediction accuracy via the ROC curve and the C-index in TCGA dataset (Figure 8, Supplementary Table 5).

Meanwhile, 50% of the samples were randomly taken for 1000 times to construct serial validation datasets for testing all the models (Supplementary Table 5). The AUC of all other prognostic biomarkers was significantly (*P* < 0.001, by *t*-test) lower than that of the RNA prognostic biomarker model (0.793, 95% CI of 0.728-0.858). These results suggested that the average AUC of our model was significantly higher than that of the others, indicating good robustness. On the other hand, the C-index of our model was also significantly superior to 20 of the other 23 markers in the references (*P* < 0.05, by *t*-test, Supplementary Table 5), and 2 of the remaining 3 markers have a lower C-index than our model. Only the C-index value of hsa-mir-3660 was higher than our RNA prognostic biomarker model, but the Kaplan-Meier analysis showed that hsa-mir-3660 cannot distinguish significantly between the high- and low-risk groups (*P* = 0.307).

In conclusion, the results demonstrated the superiority of the RNA prognostic biomarker model over other prognostic biomarkers or their combinations in accurately predicting the survival of patients.

## 4. Discussion

Drug therapy is a relatively important part of the HCC treatment strategy, but adverse reactions often occur. For example, sorafenib is a successful drug used for HCC treatment. It works through the inhibition of angiogenesis to suppress tumor progression, which remarkably declines the HCC recurrence, and thus improves the HCC prognosis. However, recent studies on sorafenib have reported elevated risks for cardiovascular and thrombotic diseases [11]. Hence, it is expected that the prognostic biomarker model proposed in this study could reduce the abuse of anticancer drugs and in turn improve the quality of treatment.

Three genes consisted in the model are ENSG00000238121, ENSG00000107937, and ENSG00000124731, respectively. *GTPBP4*, also termed as ENSG00000107937, is a member of the *G* protein family [52]. In the case of mutation, its function is deactivated, giving rise to the constantly activated state of the *RAS* protein that promotes tumor initiation and development. For example, Nissan et al. demonstrated that the *GTPBP4* mutation enhances the proliferation of melanoma cells, while the introduction of the wild-type counterpart suppresses the same [53]. Furthermore, breast cancer patients with a high expression of *GTPBP4* exhibited a short survival time [54]. Shen et al. proposed that the *GTPBP4* expression levels are associated with clinical stages, tumor metastasis, and postoperative survival time in patients with bladder urothelial carcinoma [55]. Moreover, the low expression of genes encoding the *GTP*-binding proteins was correlated with tumor stages and distal organ metastasis in patients with gastric cancer [56].

*TREM*-*1*, also known as ENSG00000124731 (the triggering receptor expressed on myeloid cells-1), was initially identified by Bouchon et al. It is an activated receptor of the TREM family, primarily expressed on the surface of neutrophils and *CD14+* monocyte/macrophage [57]. Other studies demonstrated that a high expression of *TREM*-*1* is closely associated with tumorigenesis. It mediates cell hyperplasia, thus enhancing mutation probability and ultimately giving rise to the tumor [58]. Also, its expression level was correlated with lymph node metastasis, tumor size, and vascular invasion potential [59]. Moreover, *TREM*-*1* could impact various cytokines, such as *IL*-*8*, *MCP*-*1*, and *TNF*-*α*, in the tumor microenvironment. These are also involved in tumorigenesis, invasion, and metastasis via the mechanisms of impairment and repair suppression, thereby affecting patient prognosis. For example, Ho et al. deduced that the upregulation of *TREM*-*1* expression in cultured lung cancer cells enhances the tumor cell invasion potential via mediating the bulky expressions of *NF*-*κB* and *TNF*-*α*, and the expression inhibition of *TREM*-*1* by shRNA evidently reduced the cell invasion ability of the tumors [60].

*LINC00426*, also known as ENSG00000238121, is a long intergenic non-protein-coding RNA, located in the critical region of the 13q12.3 microdeletion syndrome [61]. Unfortunately, the study on LINC00426 was limited. The Expression Atlas database (http://www.ebi.ac.uk/gxa) [62] has shown a differential expression of LINC00426 in multiple cancers. For example, LINC00426 was found to be upregulated in TNBC, non-TNBC, and HER2-positive breast cancer patients [63] and downregulated in patients with colorectal carcinoma, non-small-cell lung carcinoma [64], and lung squamous cell carcinoma [65]. Notably, LINC00426 showed to be downregulated in hepatocellular carcinoma in this database, which was consistent with our findings. However, the function of this lncRNA is unknown. Thus, the ENSG00000238121 gene may play a role in the occurrence and development of cancer, but its specific function in cancer remains to be discovered by further research.

The process of immune cells from the blood into the tumor tissue to perform functions has been termed tumor immune cell infiltration. The immune infiltration level was reported to be very important for the growth and development of tumors [66] and represented a strong positive correlation with the expression level of three biomarkers in this study. This suggested that the individual differences in the expression level of these three genes may be caused by the characteristic immune infiltration level in each patient. However, the uniqueness of this thesis has yet to be further studied. Furthermore, the functional enrichment analysis showed that the related genes were enriched in multiple pathways related to antigen-specific immunity, suggesting that these genes may be involved in the physiological processes of recognizing and killing tumor cells of the immune system.

The concentration of immune cells of different kinds in the tumor was also closely associated with patient prognosis. In this study, it was found that the infiltration level was positively correlated with all three markers. However, the expression level of only two of these markers was positively correlated with patient risk, while the remaining one shows the opposite relationship. There are two possible reasons for this paradox. The one is that immune infiltration may not be the only factor affecting the expression level of these three genes. The other one is that the relationship between immune infiltration and prognosis in HCC patients may be anfractuous. This complexity has already been suggested in other studies. For example, macrophage infiltration leads to poor outcomes in thyroid and breast cancers but improves the survival time in colorectal cancer patients. In renal cancer, neutrophil infiltration is a high-risk indicator, while the large number of the emergence of CD4+ T-cells is a low-risk signature. Notably, it has been suggested that neutrophil infiltration is detrimental to prognosis in colorectal cancer, but the opposite conclusion has been proposed in another research. In hepatocellular carcinoma, more research is needed to determine the specific mechanism of immune cell infiltration affecting prognosis, and the three genes proposed in this research may provide some insights into this area.

## 5. Conclusions

In this study, multivariate Cox regression was used to construct a prognostic biomarker model of hepatocellular carcinoma consisting of two protein-coding genes (GTPBP4, TREM-1) and one lncRNA (LINC00426). The AUC values of the model were 0.819 (95% CI of 0.730-0.908, *P* < 0.001) and 0.778 (95% CI of 0.694-0.861, *P* < 0.001) in TCGA training and validation datasets, while the C-index values in those two datasets were 0.760 (95% CI of 0.692-0.828, *P* < 0.001) and 0.685 (95% CI of 0.622-0.749, *P* < 0.001). In the GEO validation dataset, we only found the expression of two markers in the combination, but the AUC value was 0.641 (95% CI of 0.561-0.721, *P* = 0.003), and the C-index was 0.592 (95% CI of 0.538-0.647, *P* < 0.001). Meanwhile, the model also performed well in different subsets divided by gender, stage, and grade. It can be concluded that this model has good prediction performance and can effectively distinguish the high-risk and low-risk patients of hepatocellular carcinoma. Additionally, prognostic biomarkers were associated with immune infiltration, cell proliferation, invasion, and metastasis. It is expected that the model may provide a certain reference for the treatment of cancer.

## Figures and Tables

**Table 1:** Summary of HCC patients' clinicopathological characteristics from TCGA database.

Characteristics	Patient					
	Training set		Validation set		Sum	
	Amount	%	Amount	%	Amount	%
Age at diagnosis						
≥60	98	52.40	103	56.28	201	54.32
<60	89	47.60	80	43.72	169	45.68
Gender						
Male	133	71.12	116	63.39	249	67.30
Female	54	28.88	67	36.61	121	32.70
Clinical stage						
Stage I	76	40.64	95	51.91	171	46.22
Stage II	45	24.07	40	21.85	85	22.97
Stage III	47	25.13	38	20.77	85	22.97
Stage IV	4	2.14	1	0.55	5	1.35
Others/unknown	15	8.02	9	4.92	24	6.49
Tumor grade						
G1	26	13.90	29	15.85	55	14.87
G2	91	48.66	86	47.00	177	47.84
G3	63	33.69	58	31.67	121	32.70
G4	6	3.21	6	3.28	12	3.24
Others/unknown	1	0.54	4	2.20	5	1.35
Survival status						
Survive	133	71.12	107	58.47	240	64.87
Death	54	28.88	76	41.53	130	35.13

**Table 2:** Information of the 3 RNAs in the optimal prognostic biomarker model obtained via multivariate Cox regression.

Ensembl ID	Gene symbol	Chr	Coordinate	Coefficient^a^	*P* value^b^
ENSG00000107937	GTPBP4	Chr10	988409-1017771	0.7675	8.8 *e* − 04
ENSG00000124731	TREM-1	Chr6	41267385-41286745	0.1726	4.0 *e* − 04
ENSG00000238121	LINC00426	Chr13	30340266-30373914	-0.2466	1.2 *e* − 04

Notes: ^a^the coefficient value of the gene in the prognostic model of 3 RNAs derived from the multivariate Cox proportional-hazards regression analysis; ^b^the Wald test *P* value in the multivariate Cox proportional-hazards regression analysis.

## Data Availability

This study used public data accessible in The Cancer Genome Atlas database and the National Center for Biotechnology Information Gene Expression Omnibus database.

## Supplementary Materials

Supplementary Materials Supplementary Figure 1: Kaplan-Meier and the ROC curves in different clinical stages. Supplementary Figure 2: Kaplan-Meier and the ROC curves in different pathological grades. Supplementary Figure 3: Kaplan-Meier and the ROC curves in the GEO validation dataset. Supplementary Figure 4: histogram of differential expressions of the 3 RNA prognostic biomarkers in different clinical stages. Supplementary Figure 5: the scatter plot of immune cell infiltration analysis. Supplementary Table 1: the accession codes of TCGA of the 377 HCC specimens. Supplementary Table 2: Kaplan-Meier, ROC, and concordance index results of different regrouping methods. Supplementary Table 3: the subcellular localization information of 3 prognostic biomarkers. Supplementary Table 4: the list of seventeen enriched pathways related to genes that were coexpressed with 3 biomarkers. Supplementary Table 5: the bootstrap method calculating the AUC and the concordance index values to test the performance of all models. Supplementary Source code: ROC analysis source code. A new Perl script which was a lot more efficient to complete traversing all possible combinations and calculating the area under the receiver operating characteristic curve (AUC).
