# Supplementary material for: An Integrating Immune-Related Signature to Improve Prognosis of Hepatocellular Carcinoma
Source: Comput Math Methods Med. 2020 Nov 3;2020:8872329. doi: 10.1155/2020/8872329 (PMC7655255; doi:10.1155/2020/8872329)
Supplement: Supplementary Materials — Supplementary Figure 1: Kaplan-Meier and the ROC curves in different clinical stages. Supplementary Figure 2: Kaplan-Meier and the ROC curves in different pathological grades. Supplementary Figure 3: Kaplan-Meier and the ROC curves in the GEO validation dataset. Supplementary Figure 4: histogram of differential expressions of the 3 RNA prognostic biomarkers in different clinical stages. Supplementary Figure 5: the scatter plot of immune cell infiltration analysis. Supplementary Table 1: the accession codes of TCGA of the 377 HCC specimens. Supplementary Table 2: Kaplan-Meier, ROC, and concordance index results of different regrouping methods. Supplementary Table 3: the subcellular localization information of 3 prognostic biomarkers. Supplementary Table 4: the list of seventeen enriched pathways related to genes that were coexpressed with 3 biomarkers. Supplementary Table 5: the bootstrap method calculating the AUC and the concordance index values to test the performance of all models. Supplementary Source code: ROC analysis source code. A new Perl script which was a lot more efficient to complete traversing all possible combinations and calculating the area under the receiver operating characteristic curve (AUC). [file 8872329.f1.zip › Supplementary_Material(Figure_Table).pdf]

Supplementary Material

——Supplementary Figure and Table

An integrating immune-related signature to improve prognosis of hepatocellular carcinoma

Rui Zhu,[1,2] Wenna Guo,[3] Xin-Jian Xu,[1] and Liucun Zhu[2]

[1] Department of Mathematics, Shanghai University, Shanghai, 200444, China.
[2] School of Life Sciences, Shanghai University, Shanghai, 200444, China.
[3] School of Life Sciences, Zhengzhou University, Zhengzhou, Henan, 450001, China.

Correspondence should be addressed to Liucun Zhu; zhuliucun@shu.edu.cn and Xin-Jian Xu; xinjxu@shu.edu.cn

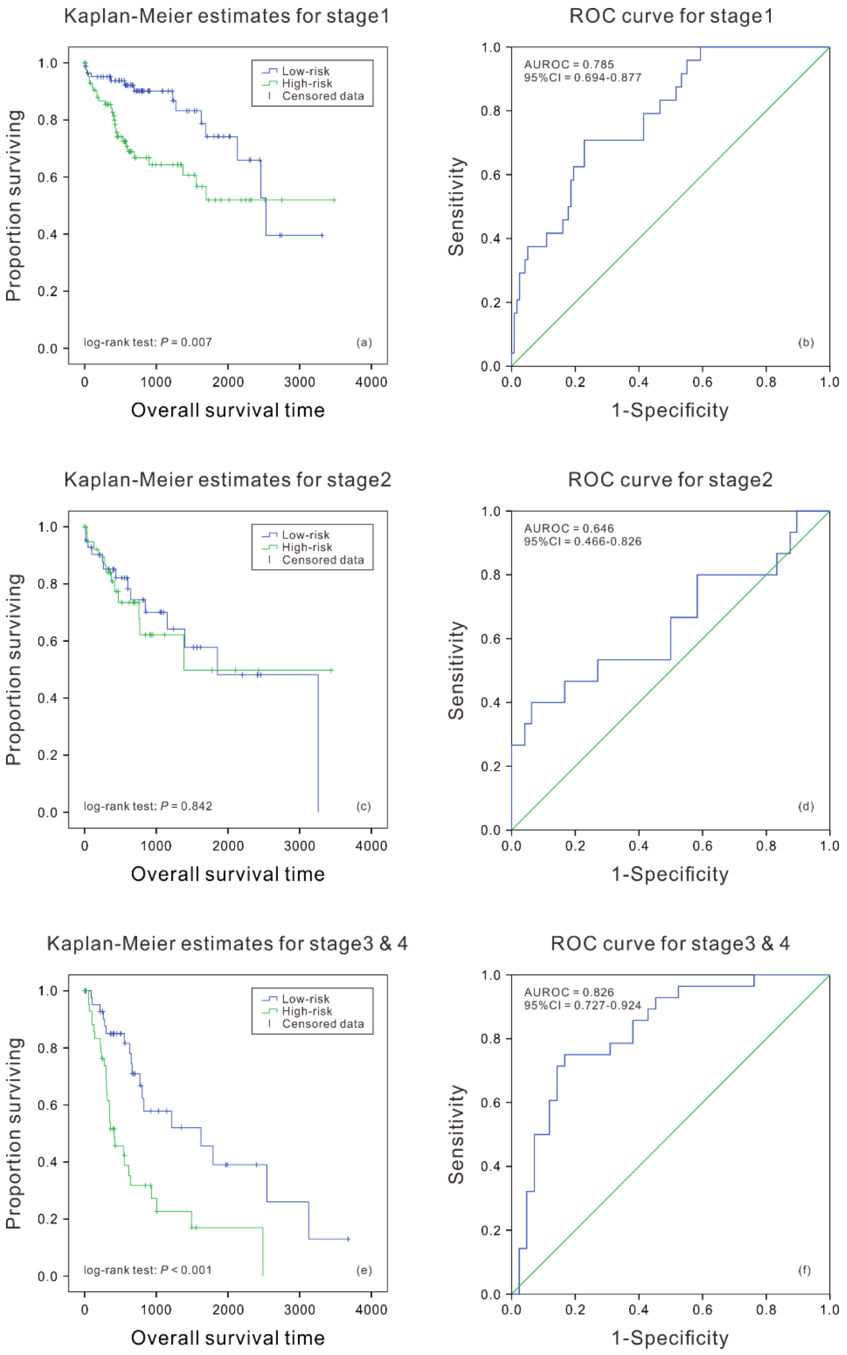

Supplementary Figure 1. Kaplan-Meier and the ROC curves in different clinical stages. It can be concluded that higher risk scores are significantly associated with overall survival time in stage 1 and stage3&4 (P = 0.007, P < 0.001). The AUC were 0.785(95% CI = 0.694-0.877) and 0.826 (95% CI = 0.727-0.927) in stage 1 and stage3&4.

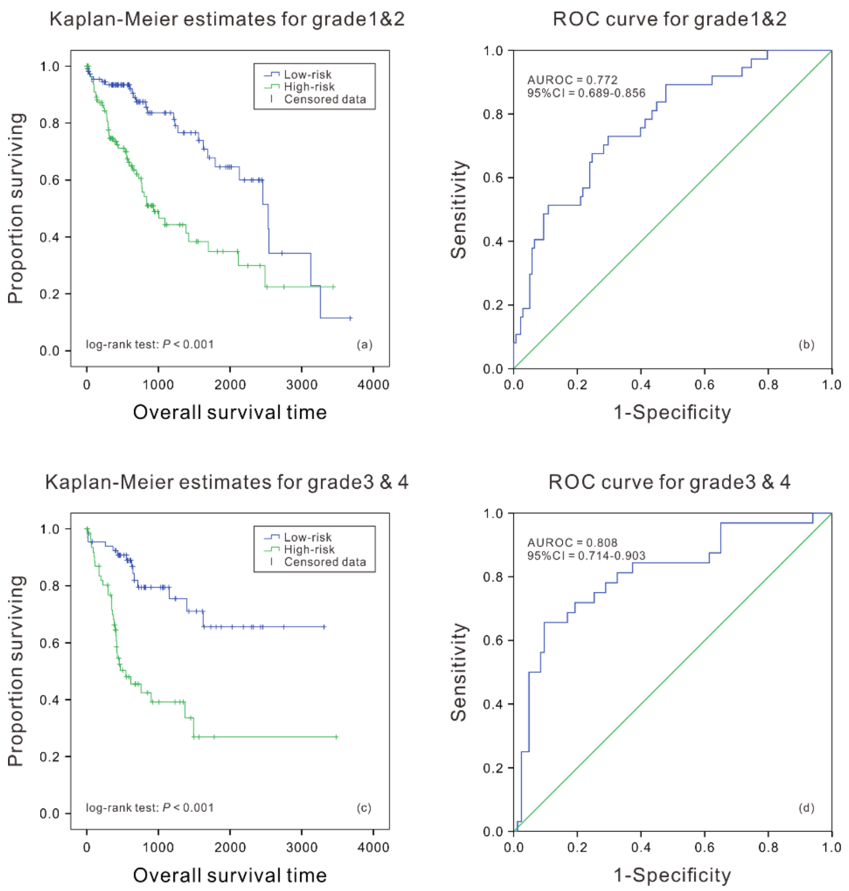

Supplementary Figure 2. Kaplan-Meier and the ROC curves in different tumor grades. It can be concluded that higher risk scores are significantly associated with overall survival time in different pathological grades (P <0.001, P < 0.001). The AUC were 0.772 (95% CI = 0.689- 0.856) and 0.808 (95% CI = 0.714-0.903).

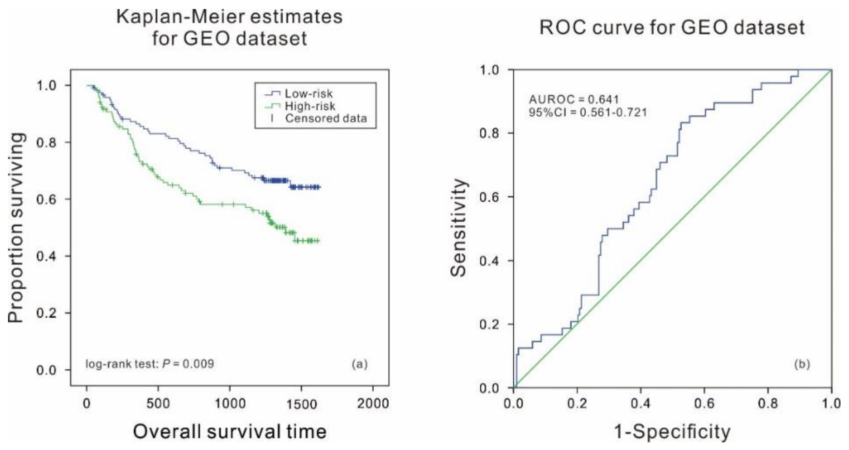

Supplementary Figure 3. Kaplan-Meier and the ROC curves in the GEO validation dataset. The results of two-sided log-rank test performed on the survival time between the low- and high-risk curves showed a significantly difference (P = 0.009) (a). ROC curve of survival prediction in the GEO validation dataset (b).

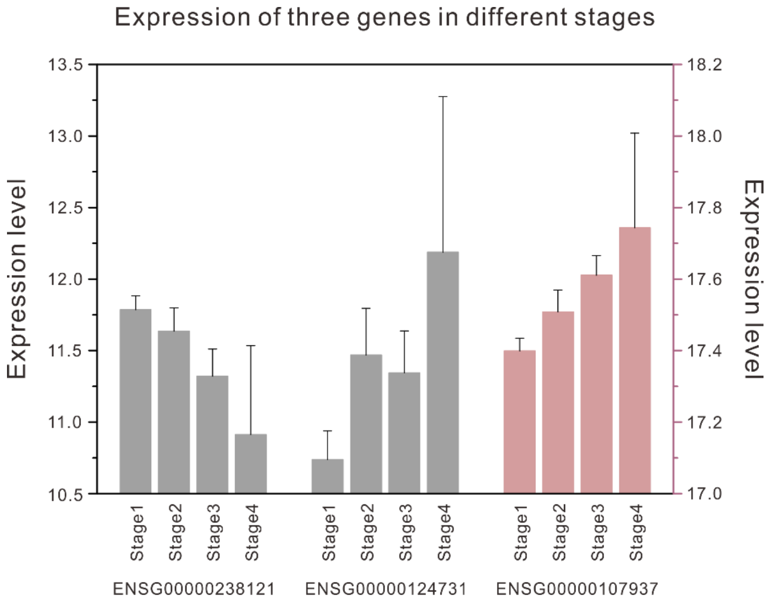

Supplementary Figure 4. Histogram of differential expressions of the 3 RNA prognostic biomarkers in different clinical stages.

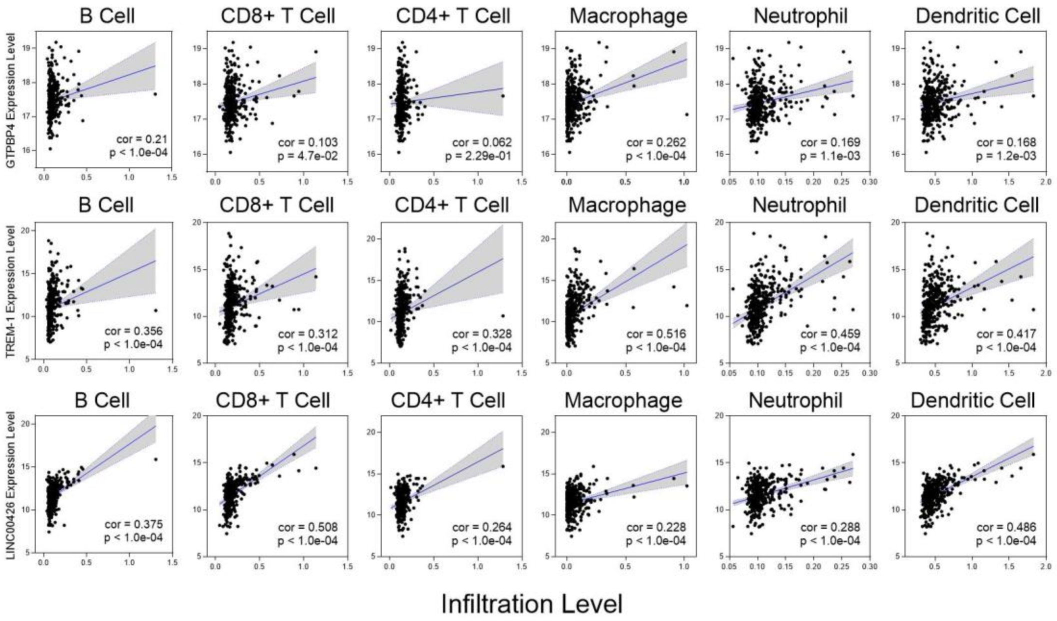

Supplementary Figure 5. The scatter plot of immune cell infiltration analysis.

Supplementary Table 1. The accession codes of TCGA of the 377 HCC specimens

Accession codes				
TCGA-RC-A6M5	TCGA-G3-A25T	TCGA-CC-A8HU	TCGA-DD-AAVQ	TCGA-DD-AAW2
TCGA-MI-A75I	TCGA-DD-AACK	TCGA-DD-A1E9	TCGA-5C-A9VG	TCGA-G3-A5SK
TCGA-RC-A7S9	TCGA-BD-A2L6	TCGA-CC-A8HV	TCGA-GJ-A3OU	TCGA-CC-A3MC
TCGA-G3-A5SJ	TCGA-RG-A7D4	TCGA-2Y-A9H1	TCGA-BW-A5NQ	TCGA-DD-AAVP
TCGA-ED-A7XP	TCGA-DD-AADN	TCGA-BC-A10R	TCGA-DD-AAE3	TCGA-DD-AAD5
TCGA-DD-AAE9	TCGA-G3-A6UC	TCGA-XR-A8TF	TCGA-ZS-A9CG	TCGA-K7-AAU7
TCGA-DD-AACI	TCGA-DD-A11C	TCGA-G3-AAV4	TCGA-CC-A7II	TCGA-G3-A3CH
TCGA-3K-AAZ8	TCGA-DD-AADE	TCGA-CC-A8HT	TCGA-DD-A4NJ	TCGA-LG-A9QC
TCGA-T1-A6J8	TCGA-G3-A3CI	TCGA-DD-AACZ	TCGA-BC-A3KG	TCGA-DD-AACS
TCGA-G3-AAUZ	TCGA-G3-A7M5	TCGA-DD-A4NG	TCGA-DD-AAD8	TCGA-DD-A1EH
TCGA-KR-A7K7	TCGA-DD-A1EI	TCGA-2Y-A9H5	TCGA-DD-AACX	TCGA-DD-AACN
TCGA-DD-AAVR	TCGA-DD-A73G	TCGA-ZP-A9CV	TCGA-2Y-A9HB	TCGA-DD-AADA
TCGA-ZP-A9CY	TCGA-DD-AAEI	TCGA-UB-A7MD	TCGA-BD-A3ER	TCGA-G3-A5SM
TCGA-G3-A25U	TCGA-XR-A8TC	TCGA-DD-A4NN	TCGA-ZP-A9D1	TCGA-CC-A9FW
TCGA-DD-AAW0	TCGA-CC-A9FU	TCGA-DD-A3A5	TCGA-DD-AACY	TCGA-G3-A25Z
TCGA-BD-A3EP	TCGA-UB-A7ME	TCGA-BC-4072	TCGA-G3-A7M8	TCGA-ED-A66Y
TCGA-CC-A7IJ	TCGA-DD-AADO	TCGA-DD-AACL	TCGA-ZP-A9D0	TCGA-DD-A1EF
TCGA-ZS-A9CF	TCGA-LG-A6GG	TCGA-CC-A7IE	TCGA-DD-AAW1	TCGA-CC-5263
TCGA-DD-AAD2	TCGA-DD-A4NH	TCGA-BC-A112	TCGA-DD-AACW	TCGA-BC-A10S
TCGA-DD-AACP	TCGA-DD-A73C	TCGA-NI-A4U2	TCGA-DD-A73D	TCGA-HP-A5MZ
TCGA-DD-AADY	TCGA-DD-AAD6	TCGA-2Y-A9GY	TCGA-2Y-A9H4	TCGA-GJ-A9DB
TCGA-DD-AACA	TCGA-DD-AADB	TCGA-2Y-A9GT	TCGA-DD-AAVY	TCGA-PD-A5DF
TCGA-WQ-A9G7	TCGA-RC-A7SF	TCGA-DD-AACG	TCGA-DD-AAD1	TCGA-UB-A7MF
TCGA-DD-A73E	TCGA-CC-A7IH	TCGA-DD-A3A4	TCGA-KR-A7K8	TCGA-DD-A39W
TCGA-ED-A8O5	TCGA-CC-A9FV	TCGA-CC-A1HT	TCGA-DD-AADU	TCGA-ZP-A9D2
TCGA-UB-A7MC	TCGA-WJ-A86L	TCGA-BC-A5W4	TCGA-DD-AACO	TCGA-DD-A4NQ
TCGA-5C-AAPD	TCGA-K7-A5RF	TCGA-CC-A5UE	TCGA-DD-AAE2	TCGA-CC-A8HS
TCGA-FV-A4ZQ	TCGA-DD-AAVS	TCGA-DD-A39Y	TCGA-DD-A73F	TCGA-FV-A3R2
TCGA-DD-AADJ	TCGA-BC-A69I	TCGA-ES-A2HS	TCGA-DD-AACJ	TCGA-DD-A1EL
TCGA-DD-AADQ	TCGA-G3-AAV0	TCGA-KR-A7K0	TCGA-UB-AA0U	TCGA-CC-A7IK
TCGA-BC-A3KF	TCGA-DD-AAEE	TCGA-BC-A110	TCGA-ED-A66X	TCGA-DD-A114
TCGA-MI-A75H	TCGA-RC-A7SB	TCGA-HP-A5N0	TCGA-DD-AAEG	TCGA-DD-A39X
TCGA-DD-AAE1	TCGA-G3-A7M7	TCGA-DD-A1EG	TCGA-XR-A8TD	TCGA-EP-A2KB
TCGA-DD-AAEK	TCGA-DD-A4NF	TCGA-DD-A115	TCGA-ED-A7XO	TCGA-DD-A39Z
TCGA-FV-A495	TCGA-ED-A459	TCGA-4R-AA8I	TCGA-WX-AA44	TCGA-DD-A3A8
TCGA-MI-A75C	TCGA-G3-A5SL	TCGA-DD-A3A6	TCGA-2Y-A9GU	TCGA-CC-5262
TCGA-2Y-A9H0	TCGA-QA-A7B7	TCGA-DD-A3A2	TCGA-DD-A1EC	TCGA-2Y-A9GV
TCGA-DD-AAE6	TCGA-ZS-A9CE	TCGA-DD-A4NK	TCGA-ED-A97K	TCGA-2Y-A9HA
TCGA-DD-AAVW	TCGA-DD-AAE4	TCGA-BC-A10Y	TCGA-BW-A5NO	TCGA-CC-A3M9

TCGA-UB-AA0V	TCGA-DD-AADK	TCGA-ED-A8O6	TCGA-DD-A4NB	TCGA-YA-A8S7
TCGA-G3-A3CG	TCGA-DD-A4NA	TCGA-DD-A4NE	TCGA-G3-A3CJ	TCGA-G3-A7M9
TCGA-DD-A73A	TCGA-ED-A627	TCGA-DD-AA3A	TCGA-DD-A118	TCGA-DD-A3A3
TCGA-MI-A75E	TCGA-DD-A1EB	TCGA-CC-5261	TCGA-BC-A8YO	TCGA-DD-A116
TCGA-G3-AAV3	TCGA-DD-A11A	TCGA-CC-A7IF	TCGA-DD-A4NO	TCGA-CC-A5UD
TCGA-K7-A5RG	TCGA-G3-AAV5	TCGA-CC-A7IG	TCGA-DD-AADR	TCGA-ES-A2HT
TCGA-DD-AADP	TCGA-DD-AAE0	TCGA-DD-AADC	TCGA-RC-A6M6	TCGA-DD-A11B
TCGA-DD-A4NL	TCGA-BC-A216	TCGA-CC-5258	TCGA-MI-A75G	TCGA-EP-A2KA
TCGA-WQ-AB4B	TCGA-K7-A6G5	TCGA-DD-A39V	TCGA-WX-AA46	TCGA-FV-A3I1
TCGA-ZP-A9CZ	TCGA-DD-AAEA	TCGA-2Y-A9GS	TCGA-XR-A8TG	TCGA-WX-AA47
TCGA-UB-A7MB	TCGA-XR-A8TE	TCGA-BC-A10T	TCGA-G3-AAV7	TCGA-FV-A3R3
TCGA-DD-AACV	TCGA-2Y-A9H7	TCGA-DD-A3A0	TCGA-DD-AACT	TCGA-2Y-A9GW
TCGA-DD-AAVX	TCGA-DD-AACU	TCGA-CC-5264	TCGA-DD-AAEB	TCGA-BC-A10Q
TCGA-FV-A2QQ	TCGA-DD-AACM	TCGA-GJ-A6C0	TCGA-DD-A4NP	TCGA-DD-AADM
TCGA-DD-AAVZ	TCGA-G3-A25W	TCGA-BC-A217	TCGA-EP-A26S	TCGA-5R-AAAM
TCGA-O8-A75V	TCGA-BC-A69H	TCGA-DD-AACQ	TCGA-DD-A4NV	TCGA-G3-AAV1
TCGA-RC-A6M3	TCGA-2V-A95S	TCGA-DD-A1EK	TCGA-DD-AACE	TCGA-DD-A11D
TCGA-ED-A7PY	TCGA-EP-A3JL	TCGA-CC-A7IL	TCGA-ED-A7PZ	TCGA-DD-A3A9
TCGA-DD-AAED	TCGA-2Y-A9H2	TCGA-CC-5260	TCGA-BW-A5NP	TCGA-BC-A10Z
TCGA-DD-A4ND	TCGA-DD-AAW3	TCGA-G3-AAV6	TCGA-G3-A25X	TCGA-BC-4073
TCGA-DD-AADV	TCGA-ED-A5KG	TCGA-DD-A1EJ	TCGA-FV-A496	TCGA-ED-A7PX
TCGA-DD-AADL	TCGA-5R-AA1C	TCGA-2Y-A9H8	TCGA-DD-AADW	TCGA-CC-5259
TCGA-G3-A7M6	TCGA-MR-A8JO	TCGA-G3-A25Y	TCGA-NI-A8LF	TCGA-FV-A3I0
TCGA-DD-AADG	TCGA-ED-A4XI	TCGA-DD-AAC8	TCGA-DD-A113	TCGA-UB-A7MA
TCGA-DD-AACB	TCGA-DD-AAE8	TCGA-DD-A73B	TCGA-DD-AAVV	TCGA-2Y-A9H3
TCGA-LG-A9QD	TCGA-DD-AADD	TCGA-2Y-A9GZ	TCGA-2Y-A9H9	TCGA-KR-A7K2
TCGA-CC-A9FS	TCGA-DD-A1EA	TCGA-DD-AACC	TCGA-2Y-A9H6	TCGA-RC-A6M4
TCGA-G3-AAV2	TCGA-MR-A520	TCGA-BC-A10U	TCGA-EP-A3RK	TCGA-DD-AAVU
TCGA-CC-A123	TCGA-DD-AADS	TCGA-DD-A4NS	TCGA-ZP-A9D4	TCGA-DD-A1ED
TCGA-DD-AAC9	TCGA-5R-AA1D	TCGA-BC-A10W	TCGA-RC-A7SK	TCGA-DD-AAEH
TCGA-RC-A7SH	TCGA-DD-AACF	TCGA-DD-A4NR	TCGA-5C-A9VH	TCGA-G3-A25V
TCGA-DD-A4NI	TCGA-DD-A3A7	TCGA-FV-A23B	TCGA-DD-AAD0	TCGA-ZS-A9CD
TCGA-EP-A12J	TCGA-FV-A2QR	TCGA-G3-A25S	TCGA-2Y-A9GX	TCGA-EP-A2KC
TCGA-ED-A82E	TCGA-G3-A5SI	TCGA-DD-A3A1	TCGA-DD-AADI	TCGA-FV-A4ZP
TCGA-CC-A3MB	TCGA-CC-A3MA	TCGA-DD-AADF	TCGA-DD-AAE7	TCGA-BC-A10X
TCGA-DD-AACH	TCGA-DD-A1EE	TCGA-DD-A119	TCGA-DD-AAD3	TCGA-DD-AACD
TCGA-G3-A3CK	TCGA-CC-A5UC			

Supplementary Table 2. Kaplan-Meier, ROC and Concordance Index results of different regrouping methods

Regrouping factor	groups	Kaplan–Meier Pvalue	AUC	AUC 95% confidence interval	Pvalue of AUC	C-Index	95% CI of C-Index	Pvalue of C-Index
**Gender**								
	Male	<0.001	0.802	0.726-0.877	<0.001	0.733	0.673-0.794	<0.001
	Female	0.038	0.754	0.645-0.863	<0.001	0.646	0.568-0.723	<0.001
**Clinical stage**								
	stage I	0.007	0.785	0.694-0.877	<0.001	0.727	0.651-0.804	<0.001
	stage II	0.842	0.646	0.466-0.826	0.09	0.589	0.465-0.712	0.1590319
	stage III&IV	<0.001	0.826	0.727-0.924	<0.001	0.709	0.637-0.782	<0.001
**Tumor grade**								
	G1&2	<0.001	0.772	0.689-0.856	<0.001	0.695	0.632-0.759	<0.001
	G3&4	<0.001	0.808	0.714-0.903	<0.001	0.727	0.653-0.8	<0.001

Supplementary Table 3. The Subcellular Localization information of 3 prognostic biomarkers

Symbol	Category	Tissue	Subcellular Localization	Validated Method/ Database	Prediction	Tool
GTPBP4	mRNA	Myeloma cell	Cytosol	qRT-PCR	Cytosol/Cytoplasm	iLoc-mRNA
		Colon cancer cell	Nucleus	NGS		
		HEK293 cell	Ribosome	NGS		
		K562	Chromatin	CSCD		
			Cytosol	CSCD		
			Nucleolus	CSCD		
			Nucleoplasm	CSCD		
		HepG2	Cytosol	CSCD		
		HeLa-S3	Nucleus	CSCD		
		Blood	Exosome	exoRBase		
TREM-1	mRNA	Blood	Exosome	exoRBase	Cytosol/Cytoplasm	iLoc-mRNA
LINC00426	LncRNA	Blood	Exosome	exoRBase	Cytosol/Cytoplasm	iLoc-LncRNA

Supplementary Table 4. The list of seventeen enriched pathways related to genes that were co-expressed with 3 biomarkers from RNA prognostic biomarkers combination for liver cancer.

Pathway	Number of genes	Fold Enrichment	P value*
T cell receptor signaling pathway	10	14.9	1.50E-06
Natural killer cell mediated cytotoxicity	9	11.3	5.20E-05
T Cytotoxic Cell Surface Molecules	5	32.2	2.60E-04
Lck and Fyn tyrosine kinases in initiation of TCR Activation	5	35.2	3.50E-04
NO2-dependent IL 12 Pathway in NK cells	5	22.8	8.00E-04
Activation of Csk by cAMP-dependent Protein Kinase Inhibits Signaling through the T Cell Receptor	5	17.6	1.80E-03
Primary immunodeficiency	5	22.6	2.50E-03
Cytokine-cytokine receptor interaction	9	6	2.80E-03
T Helper Cell Surface Molecules	4	25.8	3.60E-03
Chemokine signaling pathway	8	6.6	4.00E-03
IL 17 Signaling Pathway	4	20.6	6.30E-03
The Co-Stimulatory Signal During T-cell Activation	4	16.3	1.10E-02
IL12 and Stat4 Dependent Signaling Pathway in Th1 Development	4	14.7	1.40E-02
Hematopoietic cell lineage	5	9	3.20E-02
Cell adhesion molecules (CAMs)	6	6.5	3.60E-02
HIV Induced T Cell Apoptosis	3	23.2	4.00E-02
Fc gamma R-mediated phagocytosis	5	9.1	4.10E-02

*P value by Benjamini–Hochberg corrections method

Supplementary Table 5. The bootstrap method calculating the AUC and the Concordance Index values to test the performance of all models

	Mean of AUC	95% CI of AUC	P value of AUC*	C-Index	95% CI of C-Index	P value of C-Index*
**Our Model**	0.793	0.728-0.858	/	0.685	0.622-0.749	/
**3-gene Model**	0.76	0.695-0.825	<0.001	0.676	0.610-0.743	3.90E-01
**45-gene Model**	0.613	0.533-0.693	<0.001	0.458	0.387-0.529	3.61E-05
**ENSG00000086991**	0.539	0.472-0.606	<0.001	0.497	0.426-0.569	3.52E-05
**ENSG00000137857**	0.543	0.472-0.614	<0.001	0.482	0.414-0.55	9.03E-06
**ENSG00000160867**	0.541	0.468-0.614	<0.001	0.563	0.496-0.631	2.53E-03
**ENSG00000140443**	0.547	0.466-0.628	<0.001	0.493	0.415-0.571	6.22E-05
**ENSG00000234912**	0.566	0.486-0.646	<0.001	0.52	0.443-0.597	2.43E-04
**ENSG00000237438**	0.643	0.566-0.720	<0.001	0.597	0.524-0.67	2.93E-02
**ENSG00000255874**	0.639	0.562-0.716	<0.001	0.611	0.543-0.68	4.51E-02
**ENSG00000234608**	0.575	0.503-0.647	<0.001	0.537	0.469-0.605	4.92E-04
**hsa-mir-9-3**	0.629	0.538-0.720	<0.001	0.586	0.517-0.654	1.25E-02
**hsa-mir-10b**	0.579	0.500-0.658	<0.001	0.564	0.498-0.631	7.39E-03
**hsa-mir-31**	0.506	0.374-0.638	<0.001	0.532	0.447-0.616	1.01E-03
**hsa-mir-519c**	0.478	0.305-0.651	<0.001	0.527	0.404-0.65	1.08E-02
**hsa-mir-522**	0.47	0.332-0.608	<0.001	0.612	0.505-0.718	1.12E-01
**hsa-mir-3660**	0.44	0.388-0.492	<0.001	0.721	0.601-0.841	7.19E-01
**hsa-mir-4784**	0.558	0.429-0.687	<0.001	0.524	0.420-0.629	2.24E-03
**hsa-mir-326**	0.579	0.487-0.671	<0.001	0.574	0.499-0.649	8.01E-03

**hsa-mir-3677**	0.657	0.582-0.732	<0.001	0.609	0.535-0.683	2.37E-02
**hsa-mir-511**	0.53	0.461-0.599	<0.001	0.488	0.413-0.564	3.64E-05
**hsa-mir-9-1**	0.626	0.536-0.716	<0.001	0.586	0.516-0.655	1.29E-02
**hsa-mir-9-2**	0.632	0.546-0.718	<0.001	0.593	0.526-0.66	1.72E-02
**7-miRNA Model**	0.632	0.551-0.713	<0.001	0.592	0.523-0.661	1.64E-02

Notes:

*The *t*-test for the model and our model
