# Supplementary material for: An Integrating Immune-Related Signature to Improve Prognosis of Hepatocellular Carcinoma
Source: Comput Math Methods Med. 2020 Nov 3;2020:8872329. doi: 10.1155/2020/8872329 (PMC7655255; doi:10.1155/2020/8872329)
Supplement: Supplementary Materials — Supplementary Figure 1: Kaplan-Meier and the ROC curves in different clinical stages. Supplementary Figure 2: Kaplan-Meier and the ROC curves in different pathological grades. Supplementary Figure 3: Kaplan-Meier and the ROC curves in the GEO validation dataset. Supplementary Figure 4: histogram of differential expressions of the 3 RNA prognostic biomarkers in different clinical stages. Supplementary Figure 5: the scatter plot of immune cell infiltration analysis. Supplementary Table 1: the accession codes of TCGA of the 377 HCC specimens. Supplementary Table 2: Kaplan-Meier, ROC, and concordance index results of different regrouping methods. Supplementary Table 3: the subcellular localization information of 3 prognostic biomarkers. Supplementary Table 4: the list of seventeen enriched pathways related to genes that were coexpressed with 3 biomarkers. Supplementary Table 5: the bootstrap method calculating the AUC and the concordance index values to test the performance of all models. Supplementary Source code: ROC analysis source code. A new Perl script which was a lot more efficient to complete traversing all possible combinations and calculating the area under the receiver operating characteristic curve (AUC). [file 8872329.f1.zip › Supplementary_Material(Source_code).pdf]

Supplementary Material

——Supplementary Source code

An integrating immune-related signature to improve prognosis of hepatocellular carcinoma

Rui Zhu,[1,2] Wenna Guo,[3] Xin-Jian Xu,[1] and Liucun Zhu[2]

[1] Department of Mathematics, Shanghai University, Shanghai, 200444, China.
[2] School of Life Sciences, Shanghai University, Shanghai, 200444, China.
[3] School of Life Sciences, Zhengzhou University, Zhengzhou, Henan, 450001, China.

Correspondence should be addressed to Liucun Zhu; zhuliucun@shu.edu.cn and Xin-Jian Xu; xinjxu@shu.edu.cn

Supplementary Source code:

ROC analysis source code. A new Perl script which was a lot more efficient to complete traversing all possible combinations and calculating the area under the Receiver operating characteristic curve (AUC).

```
#!/usr/bin/perl
use strict;
use warnings;

use Getopt::Long;
use Pod::Usage;

use List::Util qw/sum/;

my $input_file;
my $exp_file;
my $out_file;

my $man    = 0;
my $help = 0;

GetOptions(
	'help|?'       => \$help,
	'man'             => \$man,
	'input=s'      => \$input_file,
	'exp=s'      => \$exp_file,
	'output=s'    => \$out_file,
) or pod2usage(2);

pod2usage(1) if $help;
pod2usage( -exitstatus => 0, -verbose => 2 ) if $man;

$|++;

#-------------------------------------------------------------------------
#load combinations
#-------------------------------------------------------------------------

open( COMB, "< $input_file" ) or die "cannot open combination file: $input_file\n";
my %coef;
while (my $comb_line = <COMB>){
	chomp $comb_line;
```

```
	my ($combs, $coefs) = split /\t/, $comb_line;
	$coef{$combs} = $coefs;
}
close COMB;
#-------------------------------------------------------------------------

#-------------------------------------------------------------------------
#load exp file
#-------------------------------------------------------------------------

open( EXP, "< $exp_file") or die "cannot open $exp_file $!\n";

my $sample_row = <EXP>;
my @sample_names = split /\t/, $sample_row;
shift @sample_names;

my $group_row = <EXP>;
my @groups = split /\t/, $group_row;
shift @groups;
map { $_ = 0 if $_ == 2 } @groups;

my %groups;
my $datas;
my $sample_cnt;

while ( my $row = <EXP> ){
	chomp $row;
	my @data = split /\t/, $row;
	my $name = shift @data;
	$sample_cnt = ( @data );

	my $i = 0;
	while ($i < $sample_cnt){
		unless ($groups[$i] == -1){
			$groups{$sample_names[$i]} = $groups[$i];
			$datas->{$name}->{$sample_names[$i]} = $data[$i];
		}
		$i++;
	}

}
close EXP;
#-------------------------------------------------------------------------
```

```
#-------------------------------------------------------------------------
#risk score & ROC
#-------------------------------------------------------------------------

open(OUT, "> $out_file") or die "cannot create $out_file\n";

foreach my $comb ( keys %coef ){
	my @markers = split /\+/, $comb;
	my @coefs = split /,/, $coef{$comb};

	my $i = 0;
	my %coefficient;
	while ( $i < (scalar @markers)) {
		$coefficient{$markers[$i]} = $coefs[$i];
		$i++;
	}

	my %scores;

	foreach my $sample ( keys %groups ) {
		my $score = 0;
		foreach my $m (@markers){
			$score += ( $coefficient{$m} * ($datas->{$m}->{$sample}) );
		}
		$scores{$sample} = $score;
	}

	my @samples = sort { $scores{$a} <=> $scores{$b} } keys %scores;
	$i = 0;
	while ($i < (@samples) ){
		my %temp = ( $samples[$i] => $groups{$samples[$i]} );
		my $same_k = 1;
		while ( $i+$same_k < (@samples) ){
			if( $scores{$samples[$i]} == $scores{$samples[$i+$same_k]}){
				$temp{$samples[$i+$same_k]} = $groups{$samples[$i+$same_k]};
			}else{
				last;
			}
			$same_k++;
		}

		if( $same_k > 1){
			my @temp = sort { $temp{$a} <=> $temp{$b} } keys %temp;
			splice @samples, $i, $same_k, @temp;
```

```
        }

        $i += $same_k;
    }

    my $positive_cnt = 0;
    my $rank = 1;
    my $rank_sum = 0;

    while ($rank <= (@samples)){
        if($groups{$samples[$rank-1]}){
            $positive_cnt++;
            $rank_sum += $rank;
        }
        $rank++;
    }

    my $negtive_cnt = (@samples) - $positive_cnt;
    my $auroc = ($rank_sum - $positive_cnt * ($positive_cnt+1)/2) / $negtive_cnt/
$positive_cnt;

    print OUT "$comb\t$auroc\t$coef{$comb}\n";

}

close OUT;
#--------------------------------------------------------------------------
```
